# Cryptolepine inhibits hepatocellular carcinoma growth through inhibiting interleukin-6/STAT3 signalling

**DOI:** 10.1186/s12906-021-03326-x

**Published:** 2021-06-02

**Authors:** Seth A. Domfeh, Patrick W. Narkwa, Osbourne Quaye, Kwadwo A. Kusi, Gordon A. Awandare, Charles Ansah, Alimatu Salam, Mohamed Mutocheluh

**Affiliations:** 1 West African Centre for Cell Biology of Infectious Pathogens, University of Ghana, Legon, Ghana; 2 Department of Biochemistry, Cell and Molecular Biology, School of Biological Sciences, University of Ghana, Legon, Ghana; 3 Department of Clinical Microbiology, School of Medicine and Dentistry, Kwame Nkrumah University of Science and Technology, Kumasi, Ghana; 4 Department of Immunology, Noguchi Memorial Institute for Medical Research, College of Health Sciences, University of Ghana, Legon, Ghana; 5 Department of Pharmacology, Faculty of Pharmacy and Pharmaceutical Sciences, College of Health Sciences, Kwame Nkrumah University of Science and Technology, Kumasi, Ghana; 6 Manhyia Government Hospital, Kumasi, Ghana

**Keywords:** Cryptolepine, IL-6/STAT3, Carcinogenesis, Hepatoma, Anti-cancer

## Abstract

**Background:**

Diverse signalling pathways are involved in carcinogenesis and one of such pathways implicated in many cancers is the interleukin 6/signal transducer and activator of transcription 3 (IL-6/STAT3) signalling pathway. Therefore, inhibition of this pathway is targeted as an anti-cancer intervention. This study aimed to establish the effect of cryptolepine, which is the main bioactive alkaloid in the medicinal plant *Cryptolepis sanguinolenta*, on the IL-6/STAT3 signalling pathway.

**Methods:**

First, the effect of cryptolepine on the IL-6/STAT3 pathway in human hepatoma cells (HepG2 cells) was screened using the Cignal Finder Multi-Pathway Reporter Array. Next, to confirm the effect of cryptolepine on the IL-6/STAT3 signalling pathway, the pathway was activated using 200 ng/mL IL-6 in the presence of 0.5–2 μM cryptolepine. The levels of total STAT3, p-STAT3 and IL-23 were assessed by ELISA.

**Results:**

Cryptolepine downregulated 12 signalling pathways including the IL-6/STAT3 signalling pathway and upregulated 17 signalling pathways. Cryptolepine, in the presence of IL-6, decreased the levels of p-STAT3 and IL-23 in a dose-dependent fashion.

**Conclusion:**

Our results demonstrated that cryptolepine inhibits the IL-6/STAT3 signalling pathway, and therefore cryptolepine-based remedies such as *Cryptolepis sanguinolenta* could potentially be used as an effective immunotherapeutic agent for hepatocellular carcinoma and other cancers.

**Supplementary Information:**

The online version contains supplementary material available at 10.1186/s12906-021-03326-x.

## Introduction

Interleukin 6 (IL-6) is produced by several cells such as tumour cells, adipocytes, fibroblasts, macrophages, hepatocytes and endothelial cells [1, 2]. This pleiotropic cytokine interacts with its receptor (IL-6R) to form the IL-6/IL-6R complex. The complex dimerises with glycoprotein 130 (GP130), a transmembrane protein, leading to the activation of Janus kinases (JAK), which in turn phosphorylates the signal transducer and activator of transcription 3 (STAT3). The phosphorylated STAT3 (p-STAT3) homodimerises and moves into the nucleus where the homodimer binds to the promoter region of effector genes including, but not limited to, IL-23, c-Myc, Bcl-2 and HIF-1α (reviewed in [3]). In normal liver cells, IL-6 plays a vital role in the defence against infection, liver regeneration and hepatocyte homeostasis [4, 5]. However, aberrant induction of IL-6 is injurious to the liver and could lead to the development of hepatocellular carcinoma (HCC) [6, 7]. Persistent hyperactivation of the IL-6/STAT3 signalling pathway also leads to angiogenesis [8], epithelial-mesenchymal transition [9] and stemness [10] in the tumour microenvironment.

Targeting the STAT3 signalling could be a novel approach to prevent and/or treat cancers since p-STAT3 contributes to malignant transformation and progression. Some studies have shown that serum IL-6 levels are related to the tumour stage and size, metastasis and survival of colon cancer patients [11], chemotherapy efficacy for advanced pancreatic cancer [12], neoadjuvant chemoradiotherapy for oesophageal carcinoma [13] and metastasis-related morbidity of breast cancer [14]. Currently, strategies are being developed to inhibit the STAT3 signalling pathway by targeting the activation of cytokine and growth factor receptors with monoclonal antibodies or receptor antagonists. For example, the use of IL-6R super-antagonists such as Sant7 inhibited IL-6-dependent human myeloma cell growth (IC50 = 0.16 nM), as well as induced cell death as a pro-apoptotic factor through the STAT3 signalling [15].

Globally, HCC is among the most life-threatening cancers [16] because the condition causes a decline in the quality of life and normally leads to death after few months to a year from the time of diagnosis [17]. According to the GLOBOCAN 2018 Report, HCC is the sixth most common cancer and the fourth cause of cancer-related deaths worldwide [18]. Chronic viral hepatitis, mainly caused by hepatitis B and C virus (HBV and HCV), account for about 80% of HCC globally [19], and in low- and middle-income countries (LMICs), about two-thirds of HCCs are associated with HBV and/or HCV infection [20, 21]. The current interventions used in the management of HCC include targeted systemic chemotherapy, liver transplantation, surgical resection, percutaneous radiofrequency ablation, transarterial chemoembolization and radioembolization [22]. Besides the cost that is associated with these therapeutic interventions, they are unavailable in LMICs, and hence the need for other sources of therapy that are cheaper, readily accessible and culturally acceptable. Most persons living in LIMCs depend on herbal medicine for their primary care needs and hence, we believe that repurposing locally acceptable herbal products will be a complementary approach in the management of HCC and other cancers. Moreover, these medicinal plants are in abundance and easily accessible.

Cryptolepine is the main bioactive alkaloid in *Cryptolepis sanguinolenta* (Lindl.) Schlechter (Apocynaceae), a medicinal plant that is indigenous to West Africa [23], and has been reported to exhibit numerous pharmacological properties such as anti-malarial, anti-bacterial, anti-fungal, anti-hyperglycaemic [reviewed in 23], and anti-inflammatory effects in different animal models [24, 25]. Due to the potent effects of cryptolepine, *C. sanguinolenta* is used in Ghana and other West African countries for the treatment of malaria [26], and other ailments in West Africa including, but not limited to, pyrexia, jaundice, septicaemia, hypertension, tuberculosis, hepatitis and diabetes [27–29]. Cryptolepine and its derivatives have been reported to be toxic to mammalian cancer cells [30–32]; an anti-cancer effect that is exhibited by directly binding to DNA and thereby inhibiting DNA replication [30, 33]. Cryptolepine has also been reported to inhibit cancer cell growth by significantly reducing enhancers of mitochondrial biogenesis and by up-regulating p53 [30, 31].

Although some of the mechanisms of cryptolepine-associated anti-cancer properties have been reported, further studies are required to explore the anti-HCC effect of cryptolepine. Here we demonstrate the effect of cryptolepine on the IL-6/STAT3 signalling pathway.

## Materials and methods

### Chemical and reagents

The cryptolepine used in this study was donated by Professor Kwesi Mensah Boadu of the Faculty of Pharmacy and Pharmaceutical Sciences at the Kwame Nkrumah University of Science and Technology, Kumasi, Ghana. The cryptolepine was prepared to a stock concentration of 861 μM in phosphate-buffered saline, filter sterilized, aliquoted into Eppendorf tubes, wrapped with aluminium foil and stored at − 20 °C until used. Recombinant human IL-6 protein (cat # ab9627), niclosamide (cat # ab120868), STAT3 (Tyr705) In-Cell ELISA Kit (cat # ab126427) and IL-23 Human ELISA Kit (cat # ab64708) were purchased from Abcam (Cambridge, USA). Cignal Finder Reporter Array Plate (cat # CCA-901 L) and Attractene Transfection Reagent (cat # 301005) were purchased from Qiagen (Germantown, USA). Dual Luciferase Reporter Assay System (cat# E1960) was purchased from Promega (Madison, USA) and Thiazolyl blue tetrazolium bromide (MTT) powder (cat # M5655-1G) was purchased from Sigma Aldrich (St. Louis, USA). All reagents and chemicals were prepared according to the manufacturers' instructions.

### Cell culture

The human hepatoma cell line (HepG2, cat # ATCC HB-8065) was purchased from American Type Culture Collection (ATCC, Manassas, USA). The cells were maintained in Eagle’s Minimum Essential Medium (EMEM, Manassas, ATCC) that has been supplemented with 10% heat-inactivated foetal bovine serum (FBS, ATCC 30–2020), 1% MEM non-essential amino acids (ScienCell, Carlsbad, USA, cat # 0823), 100 IU/mL penicillin (Gibco Life Technologies Ltd., Paisley, UK, cat # 15140122) and 100 μg/mL streptomycin (Gibco Life Technologies Ltd., Paisley, UK, cat # 15140122), to make a complete growth medium, and were grown in 5% carbon dioxide (CO_2_) under a humidified condition at 37 °C.

### Cytotoxicity assay

The HepG2 cells were cultured at 4 × 10^4^ cells per well in 96-well plates in 100 μL complete growth medium. At 24 h post-incubation, the cells were treated with 0.5–8 μM cryptolepine or 4–32 μM niclosamide. Cell viability was assessed at 24, 48 and 72 h post-treatment using an MTT assay. A volume of 20 μL MTT solution (5 mg/mL MTT working solution in phosphate-buffered saline) was added to the wells and further incubated in 5% CO_2_ under a humidified condition at 37 °C. After 3 h, the culture supernatants were carefully removed and 130 μL absolute isopropanol was added to the wells. The plate was incubated as above for 30 min and the optical density of the colour formed was read at 595 nm using an iMark Microplate Reader (Bio-Rad, USA).

### Determination of cryptolepine effects on signal transduction pathways in HepG2 cells

The effects of cryptolepine on 45 signal transduction pathways in HepG2 cells were elucidated using the Cignal Finder Multi-Pathway Reporter Array. The reporter array has 45 inducible transcription factor-responsive constructs, encoding the firefly luciferase reporter gene, which have been dried down in duplicate wells of a 96-well plate. Also, the *Renilla* luciferase gene is included in the wells to normalise transfection efficiency. To determine the effects of cryptolepine on the signalling pathways, the constructs were reverse transfected into HepG2 cells at 8 × 10^4^ cells per well using the Attractene Transfection Reagent. At 24 h post-transfection, the cells were treated with 5 μM cryptolepine, and 24 h later, luciferase assay was performed using the Dual Luciferase Reporter Assay System following the manufacturer’s instructions. The Berthold Orion Luminometer (Berthold Detection Systems, Germany) was used in the measurement of luminescence. Cells that were not treated with cryptolepine served as negative control and used to normalise the pathway activity in the cells that were treated with cryptolepine. The control experiment was done in parallel with the main experiment.

### Determination of the effects of cryptolepine on the levels of p-STAT3 and IL-23 in the presence of IL-6

The next experiments were designed to validate the down-regulatory effect of cryptolepine on the IL-6/STAT3 signalling pathway since this pathway has been implicated in the pathogenesis of cancers including HCC [7, 34]. First, the concentration of IL-6 with optimal pathway activity was determined. The HepG2 cells were cultured at 4 × 10^4^ cells per well in 96-well plates in 100 μL complete growth medium. At 24 h post-incubation, the cells were rinsed with PBS and serum-starved for another 24 h. The cells were stimulated with 50–200 ng/mL IL-6 in serum-free medium for 24 h, and the levels of phosphorylated and total STAT3 were assessed using the STAT3 (Tyr705) In-Cell ELISA Kit following the manufacturer’s instructions. The phosphorylated STAT3 (p-STAT3) levels were normalised to the total STAT3 levels as previously done [35]. Levels of IL-23 (a responsive cytokine of the STAT3 pathway (reviewed in [36]) in culture supernatants were assessed using the IL-23 Human ELISA Kit following the manufacturer’s instructions. Next, the suppression of p-STAT3 and IL-23 was determined using niclosamide, a known inhibitor of STAT3 signalling [37]. Hence, HepG2 cells were treated with 5 and 10 μM niclosamide in the presence of 200 ng/mL IL-6 in a serum-free medium. The levels of phosphorylated and total STAT3, as well as IL-23, were assessed after 24 h. Further, to determine the effect of cryptolepine on the IL-6/STAT3 signalling pathway, HepG2 cells were treated with 0.5–2 μM cryptolepine in the presence of 200 ng/mL IL-6 in serum-free medium. The additive effect of 0.5 μM cryptolepine and 5 μM niclosamide on the IL-6/STAT3 signalling pathway was also assessed by co-treating with the alkaloid and inhibitor in the presence of IL-6 and measuring the levels of total STAT3, p-STAT3 and IL-23 after 24 h.

### Data analysis

All the experiments were conducted on three different occasions and each assay was done in duplicate or triplicate. The data were analysed using Microsoft Excel (version 2019). Cell viability was determined by the ratio of cells cultured in the presence of cryptolepine to cells cultured in the absence of cryptolepine. Reporter array data were converted into fold change based on normalised firefly luciferase activity in the cells cultured in the presence of cryptolepine to the cells cultured in the absence of cryptolepine and presented on a log_2_ scale. Pathways with fold change values that were greater than 1.5 and less than 0.7 were considered as upregulated and downregulated pathways, respectively [38]. The levels of p-STAT3/STAT3 were expressed relative to the untreated control cells. The actual protein levels of IL-23 (pg/mL) were presented. Multiple comparisons were assessed using the one-way ANOVA and Bonferroni’s test for the post-hoc analysis. In all comparisons, *p* < 0.05 was considered as statistically significant.

## Results

### Cryptolepine and niclosamide inhibit the growth of HepG2 cells

**Fig. 1:**
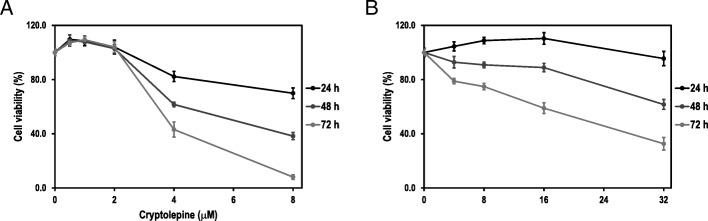
Cytotoxic effects of cryptolepine (**A**) and niclosamide (**B**) on HepG2 cells. The cells were treated with 0.5–8 μM cryptolepine or 4–32 μM niclosamide. The cell viability was assessed at 24, 48 and 72 h using an MTT-based assay. Data are presented as means and standard deviations of three independent experiments, with each experiment conducted in triplicate

Cryptolepine inhibited the growth of HepG2 cells in a dose- and time-dependent fashion (Fig. 1A). IC_50_ values at 48 and 72 h post-cryptolepine treatment were 6.8 and 3.8 μM, respectively. Similarly, niclosamide inhibited the growth of HepG2 cells in a dose- and time-time dependent fashion (Fig. 1B). The IC_50_ values at 48 and 72 h post-niclosamide treatment were 34.8 and 19.0 μM, respectively.

### Cryptolepine differentially regulates signal transduction pathways

**Fig. 2:**
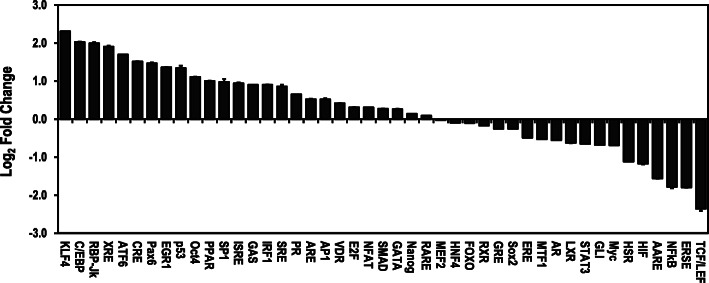
Cryptolepine differentially regulates signal transduction pathways in HepG2 cells. The cells were reversed transfected at 8 × 10^4^ cells per well in the 96-well Cignal Finder 45-Pathway Reporter Array Plate. At 24 h post-transfection, the cells were treated with a fresh medium containing 5 μM cryptolepine for 24 h. Expression of the transcription factors was measured using the dual luciferase reporter gene assay and expressed in Log_2_ of fold change. Data are presented as means and standard deviations of three independent experiments, with each experiment conducted in duplicate

The regulatory effects of cryptolepine on forty-five (45) signal transduction pathways in HepG2 cells are summarised in Fig. 2. Cryptolepine upregulated seventeen (17) signal transduction pathways (Fig. 2, S1) and downregulated twelve (12) signal transduction pathways (Fig. 2, S2). Moreover, cryptolepine did not affect sixteen (16) signal transduction pathways (Fig. 2, S3).

### p-STAT3 and IL-23 levels in the presence of IL-6 and niclosamide

**Fig. 3:**
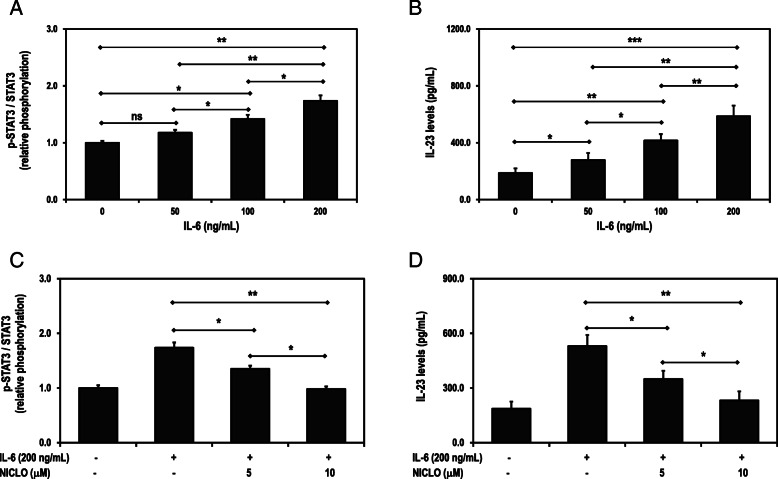
IL-6 effects on p-STAT3 (**A**) and IL-23 levels (**B**) in HepG2 cells. The cells were stimulated with IL-6 and the levels of total STAT3, p-STAT3 and IL-23 were measured after 24 h. Niclosamide (NICLO) effects on p-STAT3 (**C**) and IL-23 levels (**D**) in the presence of IL-6 in HepG2 cells. The cells were treated with niclosamide in the presence of IL-6. The levels of total STAT3, p-STAT3 and IL-23 were assessed after 24 h. Data are presented as means and standard deviations of three independent experiments, with each experiment conducted in triplicate. *p < 0.05, **p < 0.01 and ***p < 0.001 corresponds to significant difference as determined by one-way ANOVA and Bonferroni’s test, ns: difference statistically not significant

A dose-dependent increase in p-STAT3 and IL-23 levels in HepG2 cells were observed in the presence of 50–200 ng/mL IL-6 (Fig. 3A and B). In subsequent experiments, 200 ng/mL IL-6 was used to activate the IL/6-STAT3 signalling pathway. There was a decrease in p-STAT3 and IL-23 levels in the presence of 200 ng/mL IL-6 and 5–10 μM niclosamide (Fig. 3C and D). There was over 50% suppression of the p-STAT3 and IL-23 levels at 10 μM niclosamide, and hence, this concentration was used as a positive control.

### Cryptolepine suppresses the levels of p-STAT3 and IL-23 in a dose-dependent fashion in the presence of IL-6

**Fig. 4:**
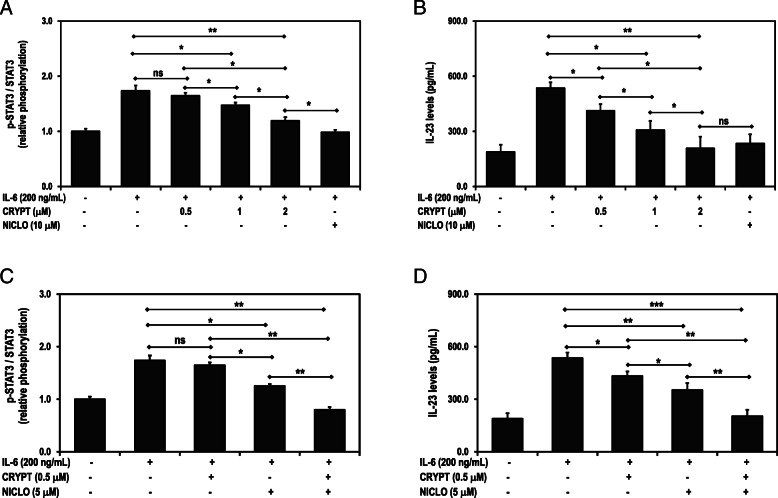
Cryptolepine (CRYPT) effects on p-STAT3 (**A**) and IL-23 levels (**B**) in the presence of IL-6 in HepG2 cells. The cells were treated with cryptolepine in the presence of IL-6. The levels of total STAT3, p-STAT3 and IL-23 were assessed after 24 h. Cryptolepine and niclosamide additive effects on p-STAT3 (**C**) and IL-23 levels (**D**) in the presence of IL-6 in HepG2 cells. The cells were co-treated with cryptolepine (CRYPT) and niclosamide (NICLO) in the presence of IL-6. The levels of total STAT3, p-STAT3 and IL-23 were assessed after 24 hours. Data are presented as means and standard deviations of three independent experiments, with each experiment conducted in triplicate. *p < 0.05, **p < 0.01 and ***p < 0.001 corresponds to significant difference as determined by one-way ANOVA and Bonferroni’s test, ns: difference statistically not significant

Cryptolepine suppressed the levels of p-STAT3 and IL-23 in a dose-dependent fashion (Fig. 4A and B) in the HepG2 cells. Moreover, cryptolepine and niclosamide additively suppressed the levels of p-STAT3 and IL-23 pathways in HepG2 cells (Fig. 4C and D).

## Discussion

Medicinal plants are chemically diverse and are resource-rich for the discovery of novel drugs [39] and hence, natural products and their structural analogues have made a key impact in pharmacotherapy, predominantly in infectious diseases as well as cancers (reviewed in [40]). Furthermore, herbal medicines are emerging as new and innovative compounds with amazing pharmacological effects that can be exploited to halt human ailments (reviewed in [41]), and one such candidate that needs to be explored is cryptolepine. Although there is limited data on the modulatory effects of cryptolepine on signal transduction pathways, few studies have delved into the cytotoxic effects of cryptolepine in vitro and its anti-cancer potential. A previous study [31] has established that cryptolepine inhibits melanoma cells in a time and dose-dependent manner, which is consistent with the findings of this current study. The mechanism of cryptolepine inhibition of melanoma cell growth has been shown to involve reducing the enhancers of mitochondrial biogenesis and the reduction of the c-Myc levels [31].

In this current study, cryptolepine up-regulated anti-cancer pathway reporters such as p53, SP1 and ISRE. Cryptolepine has been shown to impede cancer cell growth via the induction of the p53 signalling pathway [30, 32] which is consistent with the findings of this study. The p53 signalling induces apoptosis and cell cycle arrest thereby serving as a tumour suppressing pathway [42], and this accounts for the *TP53* mutation in about 50% of cancers [43]. The SP1 signalling is another pathway of interest because SP1 dysregulation has been reported in many diseases and cancers [44]. This pathway plays a role in immune responses, apoptosis, and cell growth and differentiation [45]. Overexpression of SP1 has been reported to induce p53-dependent apoptosis in tumours [46] and hence, this could be another mechanism via which cryptolepine exhibits its anti-cancer effects which need to be explored. Induction of the type 1 interferon response signalling pathway leads to antiviral, anti-cancer, and anti-inflammatory mechanisms [47–49]. Thus, the up-regulation of this multi-functional innate immune pathway is essential in immunotherapeutic strategies [50] and interestingly, cryptolepine up-regulated the reporter (ISRE) of this pathway. Currently, experiments are ongoing in our laboratory to validate the up-regulatory effect of cryptolepine on this innate immune pathway.

On the contrary, cryptolepine down-regulated pro-cancer pathway reporters such as STAT3, NF-κB and c-Myc. Chronic activation of NF-κB signalling results in the pathogenesis of numerous inflammatory and autoimmune diseases [51]. Hence, the suppression of this signalling is indispensable in the management of cancers and autoimmune diseases. The down-regulation of the NF-κB signalling in this current study is in consonance with a previous study [52]. Also in animal models, *C. sanguinolenta* and cryptolepine have been shown to suppress inflammation [24]. In over 50% of cancers, the oncogenic c-Myc is deregulated and this is often linked to unfavourable prognosis and poor patient survival [53]. The c-Myc plays a key function in oncogenesis and hence, c-Myc inhibition has been proposed as a powerful approach in cancer management [54]. The down-regulation of c-Myc as reported in this study agrees with a previous study which reported that cryptolepine reduces c-Myc levels in melanoma cells both in vitro and in vivo [31].

**Fig. 5:**
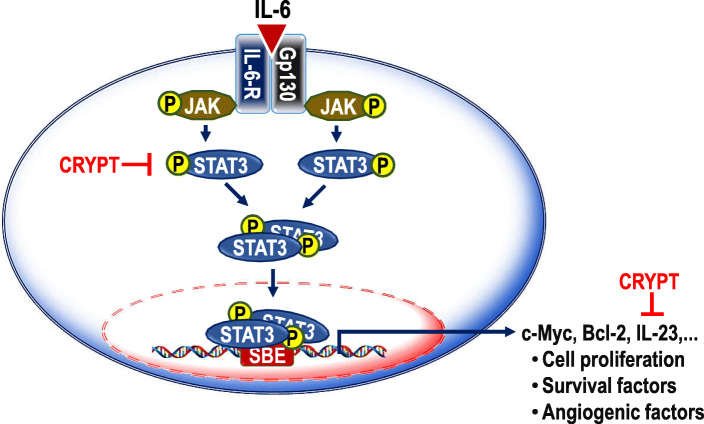
Proposed inhibition of the IL-6/STAT3 signalling pathway by cryptolepine. IL-6 interacts with IL-6R forming the IL-6/IL-6R complex. This complex then dimerises with GP130, a transmembrane protein, leading to the activation of the JAKs which in turn activate STAT3 via phosphorylation. The phosphorylated STAT3 (p-STAT3) homodimerise and translocate into the nucleus where the p-STAT3 dimers bind to the promoter regions of effector genes including, but not limited to, IL-23, c-Myc, Bcl-2 and HIF-1α. This study has demonstrated that cryptolepine suppresses the levels of p-STAT3 and IL-23 (indicated by red font and straight lines crossed at one end). CRYPT: Cryptolepine

STAT3 functions in cell differentiation, proliferation and survival. Hence within the normal hepatocytes, STAT3 signalling promotes liver regeneration, glucose homeostasis and hepatic lipid metabolism [55]. However, aberrant IL-6 mediated STAT3 signalling has been reported to suppress anti-cancer immunity in HCC [56], making the STAT3 signalling a target in cancer treatment [57, 58]. Also, studies have reported that the IL-6/STAT3 pathway activated genes arrest apoptosis, enhance angiogenesis and promote cell survival [reviewed in 3]. For example, a responsive cytokine of the IL-6/STAT3 signalling pathway, IL-23 has been reported to promote the malignant properties of hepatoma cells [34]. Also, increased levels of IL-23 have been reported to promote HCC development after chronic hepatitis B virus infection [59]. Hence, the down-regulation of the IL-6/STAT3 signalling is critical in cancer management [60, 61]. Natural products exhibit potent anticancer effects through diverse mechanisms. However, data on the therapeutic potency of phytochemicals as inhibitors of the IL-6/STAT3 signalling is limited [reviewed in 41]. According to our results, cryptolepine inhibited the IL-6/STAT3 signalling pathway by suppressing the levels of p-STAT3 and IL-23. Our result is similar to a previous study which reported that β-escin (a compound present in the medicinal plant *Aesculus hippocastanum*) induced cytotoxicity in human hepatocellular carcinoma via the suppression of STAT3 activation [62]. Similarly, diosgenin (a compound present in the medicinal plant *Trigonella foenum-graecum*) has been shown to inhibit the growth of human hepatocellular carcinoma via inhibiting both inducible and constitutive activation of STAT3 [63]. STAT3 signalling has a close association with inflammation which is subsequently linked with tumour initiation and tumorigenesis (reviewed in [64]). Hence, the suppression of the IL-6/STAT3 signalling pathway by cryptolepine (Fig. 5), as well as inflammation [24], suggest that cryptolepine could be exploited as a cheaper and novel anti-cancer agent in the management of HCC and other cancers. Also, cryptolepine and niclosamide additively inhibited the IL-6/STAT3 signalling pathway which is an interesting finding and could be further exploited as a combination therapy. Niclosamide is an FDA-approved anthelmintic drug that has been reported to inhibit the IL-6/STAT3 signalling pathway [65].

Based on the present data and previous reports, we propose that inhibition of the IL-6/STAT3 signalling pathway may promote the introduction of anti-tumour immunity into the tumour microenvironment. Moreover, enhancement of anti-cancer pathways and inhibition of pro-cancer pathways by cryptolepine may be a promising basis for the development of more effective immunotherapeutic strategies including repurposing cryptolepine-based remedies such as *Cryptolepis sanguinolenta* for the managment of hepatocellular carcinoma and other cancer patients.

## Supplementary Information

**Additional file 1: S1.** Signal transduction pathways up-regulated by cryptolepine. **S2.** Signal transduction pathways down-regulated by cryptolepine. **S3.** Signal transduction pathways unaffected by cryptolepine.

## Data Availability

The datasets used during the current study are available from the corresponding author on reasonable request.

## Abbreviations

Bcl: B-cell lymphoma
FDA: Food and drugs authority
HCC: Hepatocellular carcinoma
HIF: Hypoxia-inducible factors
IL: Interleukin
ISRE: Interferon stimulated response elements
JAK: Janus kinase
NF-κB: Nuclear factor-kappa B
SBE: STAT3 binding element
SP: Specificity protein
STAT: Signal transducer and activator of transcription
