# Supplementary material for: Cryptolepine inhibits hepatocellular carcinoma growth through inhibiting interleukin-6/STAT3 signalling
Source: BMC Complement Med Ther. 2021 Jun 2;21:161. doi: 10.1186/s12906-021-03326-x (PMC8170807; doi:10.1186/s12906-021-03326-x)
Supplement: Supplementary file 1 — Additional file 1: S1. Signal transduction pathways up-regulated by cryptolepine. S2. Signal transduction pathways down-regulated by cryptolepine. S3. Signal transduction pathways unaffected by cryptolepine. [file 12906_2021_3326_MOESM1_ESM.pdf]

Cryptolepine inhibits hepatocellular carcinoma growth through inhibiting interleukin-6/STAT3 signalling

Supplementary Data

S1: Signal transduction pathways up-regulated by cryptolepine

Reporter	Pathway	Transcription Factor	Fold Change
KLF4	KLF4	KLF4	4.94
C/EBP	C/EBP	C/EBP	4.08
RBP-Jk	Notch	RBP-Jk	3.98
XRE	Xenobiotic	AhR	3.74
ATF6	ATF6	ATF6	3.24
CRE	cAMP/PKA	CREB	2.85
Pax6	Pax6	Pax6	2.76
EGR1	EGR1	EGR1	2.57
p53	p53/DNA Damage	p53	2.53
Oct4	Oct4	Oct4	2.15
PPAR	PPAR	PPAR	2.00
SP1	SP1	SP1	1.97
ISRE	Type I Interferon	STAT1/STAT2	1.92
GAS	Interferon Gamma	STAT1/STAT1	1.87
IRF1	Interferon Regulation	IRF1	1.87
SRE	MAPK/ERK	Elk-1/SRF	1.81
PR	Progesterone Receptor	Progesterone Receptor	1.57

Differentially regulated pathways with a fold change greater than 1.5 are reported.

S2: Signal transduction pathways down-regulated by cryptolepine

Reporter	Pathway	Transcription Factor	Fold Change
MTF1	Heavy Metal Stress	MTF1	0.69
AR	Androgen Receptor	Androgen Receptor	0.68
LXR	Liver X Receptor	LXRa	0.64
STAT3	STAT3	STAT3	0.64
GLI	Hedgehog	GLI	0.63
Myc	c-myc	Myc/Max	0.62
HSR	Heat Shock Response	HSF	0.46
HIF	Hypoxia	HIF-1	0.44
AARE	Amino Acid Deprivation Response	ATF4/ATF3/ATF2	0.34
NF-κB	NF-κB	NF-κB	0.29
ERSE	Endoplasmic Reticulum Stress	CBF/NF-Y/YY1	0.29
TCF/LEF	Wnt	TCF/LEF	0.20

Differentially regulated pathways with a fold change of less than 0.7 are reported.

S3: Signal transduction pathways unaffected by cryptolepine

Reporter	Pathway	Transcription Factor	Fold Change
ARE	Antioxidant Response	Nrf2/Nrf1	1.44
AP1	MAPK/JNK	AP-1	1.43
VDR	Vitamin D Receptor	Vitamin D Receptor	1.34
E2F	Cell Cycle	E2F/DP1	1.24
NFAT	PKC/Ca^{2+}	NFAT	1.24
SMAD	TGFβ	SMAD2/SMAD3/SMAD4	1.21
GATA	GATA	GATA	1.20
Nanog	Nanog	Nanog	1.10
RARE	Retinoic Acid Receptor	Retinoic Acid Receptor	1.06
MEF2	MEF2	MEF2	0.98
HNF4	Hepatocyte Nuclear Factor 4	HNF4	0.94
FOXO	PI3K/AKT	FOXO	0.93
RXR	Retinoid X Receptor	Retinoid X Receptor	0.89
GRE	Glucocorticoid Receptor	Glucocorticoid Receptor	0.84
Sox2	Sox2	Sox2	0.84
ERE	Estrogen Receptor	Estrogen Receptor	0.70

Differentially regulated pathways with a fold change from 0.7 to 1.5 are reported.
